# Health and safety in hair salons during the Covid-19 situation: A cross-sectional study in a semi-urban district in Thailand

**DOI:** 10.1016/j.puhip.2024.100472

**Published:** 2024-01-30

**Authors:** Chonyitree Sangwijit, Fatima Ibrahim Abdulsalam, Nitikorn Phoosuwan

**Affiliations:** a Department of Community Health, Faculty of Public Health, Kasetsart University Chalermphrakiat Sakon Nakhon Campus, Sakon Nakhon Province 47000, Thailand; b Department of Public Health and Caring Sciences, Faculty of Medicine, Uppsala University, Sweden

**Keywords:** COVID-19, Hair salons, Northeastern Thailand, Public health compliance, Health and safety

## Abstract

**Objective:**

Since the COVID-19 crisis in Thailand, the need for salons to have impeccable hygiene and client-hairdresser monitoring heightened. Due to scarce research on the COVID-19 preventive measures taken by hairdressing salons in semi-urban locations in Thailand during the pandemic, this study aimed to evaluate the standard of hair salons in preventing COVID-19 disease transmission in a semi-urban district in the northeastern region of Thailand.

**Methods:**

Using the purposive sampling method, data were collected from 22 Hair Salons. Data collection tools were a self-completed questionnaire designed into different sections to obtain information on demographics, work conditions and environmental health standard compliance according to guidelines set by the Thai Ministry of Public Health during the COVID-19 pandemic. Descriptive analyses were done, such as mean, standard deviation, and frequency.

**Results:**

The mean age of our respondents was 41.82 (±8.18) years, more than half were females (63.6 %). Most of the criteria assessing beauty salon standards according to Department of Health guidelines were passed, with all of the salons passing the lighting evaluation and mostly passing the heat and electric shock protection system evaluation, but the implementation of guidelines for preventive measures during the COVID-19 epidemic, according to Department of Health guidelines, suggested non-compliance by most hair Salons.

**Conclusion:**

Beauty salons should implement and strictly adhere to guidelines according to Department of Health standards. Training or education sessions regarding the prevention of infectious disease transmission should be conducted, as hairdressers should be motivated to comply with health and environmental health standards for both salon staff and clients' confidence. Further research should also be done on the behaviours associated with health risks in beauty salons at the national or border-nation level.

## 1 Introduction

Since the outbreak of the novel coronavirus (SARS-CoV-2), transmission from person to person spread to over 200 countries and territories. A global pandemic was declared on 11 March 2020 by the WHO Director-General [1]. The disease symptoms varied from asymptomatic infections, pneumonia, influenza-like illness, respiratory distress, coagulopathy, to death [[2], [3], [4]]. At the time due to unavailable safe and effective vaccines or drugs, early measures and efforts to curb the disease spread focused on non-pharmaceutical interventions (NPIs) such as travel restrictions, wearing of masks in public places, handwashing, social distancing, school/venue closures, lockdowns or stay-at-home orders and non-essential business closures including beauty parlours [2]. At the beginning of the pandemic, the Thai government had to establish the Center for COVID-19 Situation Administration (CCSA) which was tasked with drafting and disseminating policies and guidelines with the aim to prevent COVID-19 infection to local government organizations. Policies were passed through ministries, departments and provinces, delegating each province to enforce its directives according to the pandemic situation. Thailand employed surveillance and rapid response teams (SRRTs) for contact tracing, surveillance activities, public health education and promotion of the disease, and monitoring of the infected placed in quarantine [5]. With all the strict laws in place to curb the COVID-19 disease spread, about 12,000 new cases per day were recorded during the third wave of the pandemic [5].

A beauty salon, often known as a hairdresser or hairstylist, is an establishment that sells the aesthetic beauty of the hair, face, and body by the use of cosmetic products and tools, providing a variety of services including hairdressing, massage, nail care, mud baths, and body hair removal, to mention but a few [6]. According to the Public Health Act of 1992 in Thailand, beauty salons are categorized as health hazards. This classification necessitates the implementation of measures to manage, supervise, and monitor hygiene issues for both salon staff and clients. The Bureau of Environmental Health, Department of Health, is the agency that sets assessment criteria to certify standards by allowing the municipal office to control the affairs in the area. Including workplace set standards for heat stress, illumination, and noise by the Ministry of Labour [7]. The heat stress standards use WBGT (Wet Bulb Globe Temperature) limits for light, medium, and heavy work set at 34 °C, 32 °C, and 30 °C, respectively. The minimum illumination level in the workplace should be 200 lux, and the threshold noise level should not exceed 85 dBA [7]. At work, salon workers are exposed to not only physical, chemical, and biological hazards but also experience ergonomic health hazards [8,9]. Although hairdressers (also called hairstylists) are exposed to a variety of hazards in the workplace, in the field of occupational health, the activity of professionals working in beauty parlours seems to be one of the least studied [8]. The beauty salon is considered a favourable occupation in Thailand with increasing presence, particularly in urban and municipal areas [10,11]. Although beauty salons do marvel at the prettiness of their clients, they have been considered a health concern in the spread of infectious diseases [6].

Much like countries all around the world in response to the COVID-19 crisis, government entities put policies in place to shut down businesses and social activities such as beauty salons; and once reported cases started to decline, they became partially open to mitigate economic costs while keeping the transmission under control [12]. Since the COVID-19 crisis in Thailand, the need for salons to have impeccable hygiene and client-hairdresser monitoring heightened. Hence in late September 2020, new policies and protocols involving small businesses and service providers such as salons were launched by the Thai Ministry of Public Health which include increased ventilation, 2-m social distancing, mask and glove usage, face shields, and frequent disinfection of work stations [13]. A survey conducted in Bangkok during the pandemic found that about 60 % of hairdressers remained at work although they were sick and about half of them did not undergo their annual health check-ups [14]. A study has shown that to mitigate the future spread of infectious diseases such as the COVID-19 pandemic, there is a need for more research highlighting community-based policies during stylist-client interactions [15]. Due to scarce research on the COVID-19 preventive measures taken by hairdressing salons in semi-urban locations in Thailand during the pandemic, this study aims to evaluate the compliance of guidelines and recommendations meant to prevent the spread of COVID-19 within hair salons.

## 2 Materials and methods

### 2.1 Study area

**Fig. 1:**
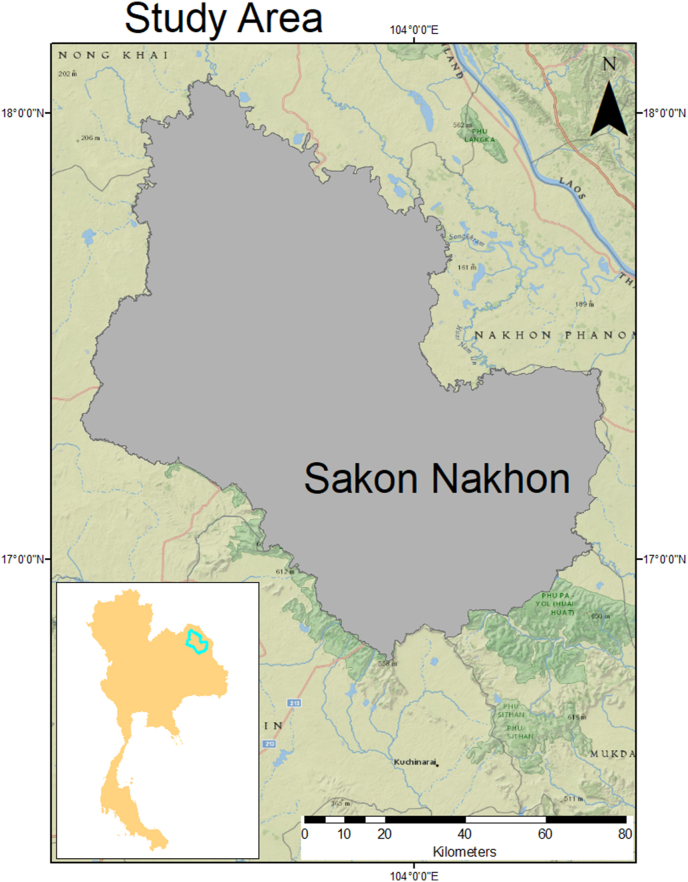
The study area shows Sakon Nakhon Province situated in northeastern Thailand. Source: Natural Earth (http://www.naturalearthdata.com/).

The study was conducted in a semi-urban sub-district in the capital city of Mueang District (situated between latitude 17°24′18″N and longitude 103°46′1.2″E) of Sakon Nakhon Province, located in the northeastern region of Thailand, where there is a combination of urban and rural areas. As such, the communities were separated into two types: traditional communities that continue country lifestyles and new communities based on University student lifestyles. With a current population of 113,722 (56,134 males and 57,588 females), a total area of ∼1496.3 km^2^ and a population density of 76.02 persons/km [2] (Fig. 1) [[16], [17], [18]]; Mueang district is the provincial centre for education, health, finance and commerce. The selected sub-district Chiang Khruea is about 20 km away from the city with a population of 15,000 people [17].

### 2.2 Data collection and data analysis

This was a cross-sectional survey aimed to evaluate the compliance of guidelines to prevent the spread of COVID-19 within beauty salons. Data were collected from July to November 2021. Areas with clusters of hair salons were visited after mapping them out in each neighbourhood using the purposive sampling method. Also known as selective or subjective sampling, purposive sampling focuses on the investigation based on the researcher's judgement. Researchers choose members of the population to participate in their study. The goal of purposive sampling is to focus on particular characteristics of a population of interest, which are best to answer the research questions. In this case, 22 beauty salon participants were recruited from 26 beauty salons with particular characteristics of interest. Beauty salons did not consent and were analyzed after questionnaires with missing answers were excluded. The research team visited the salons in person where the managerial staff (or owner) was approached on arrival at the salon, informed about the research project, assured anonymity and confidentiality and told that he/she could drop out at any time. If they agreed, other staff present were invited to complete the survey. In cases where the owners were the only staff or also working as staff, they were eligible to complete the survey. Lighting, noise, and heat stress were all evaluated by environmental health officials. Instruments used (i.e. lux meter, WBGT meter, and sound level meter) were calibrated before data collection. The Lux meter used a cut-off of 200 lux, the WBGT evaluation used a cut-off of 32 °C, and the sound level meter evaluation took a cut-off of 85 dBA.

Data collection tool was provided in Thai, and instructions were given on how to fill out the questionnaire. A three-page self-completed questionnaire, using a close-ended question and including an information page describing the study was distributed, and all respondents were incentivized to participate in the study. The questionnaire for the data collection was developed in three sections. The first section had questions on general information, the second section had questions to assess the standards of the salon and the last section was on the preventive measures done to curb the spread of the COVID-19 infection. The self-applied questionnaire was designed into different sections to obtain information on demographics, health and safety practices, years of service and/or training, work conditions (such as work environment or posture, lighting, noise, temperature, equipment, tool or furniture quality) and environmental health standard compliance according to the guidelines set by the Thai Ministry of Public Health during the COVID-19 pandemic [19].

To certify the standard of the beauty salons, assessments were carried out according to the set guidelines. The standard assessment form has a list of benchmark items where each is graded as **“pass”** or **“fail”**. A pass is given, and **“✓”** is ticked if the condition is standard and correct according to the assessment guideline; a fail is given, and **“x”** is ticked if the condition does not meet the assessment guideline [19]. This was also done to record the preventive measures implemented by the salons during the pandemic to curb the spread of the COVID-19 disease. The questionnaire was tested for content validity and reliability. Experts were consulted to score the research tool by evaluating the item validity (Item-Objective Congruence (IOC)) based on the score range from −1 to +1. Questions with IOC values ranging from 0.50 to 1.00 were valid and used, while questions with IOC values lower than 0.50 were revised. The reliability of the questionnaire was determined to ensure that the responses collected by questionnaire were reliable and consistent. The questionnaire was tested in five beauty salons in an entirely different location, not included in the sample group. The reliability value was calculated by using Cronbach's alpha [20] to ensure there was internal consistency within the items. The coefficient value of Cronbach's Alpha was more than 0.7, indicating our research tool was reliable.

To analyse the data, descriptive statistics were performed by using the SPSS program v21 (SPSS Inc., Chicago, USA) and presented as the mean and standard deviation for quantitative data, and as frequencies and per cent values for categorical data.

### 2.3 Ethics statement

Ethical approval for the study protocol was obtained from the Ethics Committee of Kasetsart University with the approved number KUCSC-HE-62-015. All the information obtained was guaranteed anonymous and participants’ privacy and confidentiality during and following completion of the study were duly ensured.

## 3 Results

The general characteristics of the participants (22 recruited; one beauty salon per questionnaire) are presented in Table 1. The majority of the respondents were female (63.6 %) in the age group 41–50 years (45.5 %). The most commonly acquired level of education was primary/elementary school (45.5 %) and service years of one to five (45.5 %). Most of the respondents (55.5 %) did not have a business license. The types of services rendered (be it a female, male, or unisex salon) were almost equivalent. Most beauty salons had only one worker (95.0 %), with no assistant (91.0 %).

**Table 1:** General characteristics of beauty salon workers (n = 22).

Characteristics	n	%
Gender		
Female	14	63.6
Male	8	36.4
Age (years)		
≤30	3	13.6
31–40	6	27.3
41–50	10	45.5
>50	3	13.6
Mean ± SD (Range) = 41.82 ± 8.18 (26.0–56.0)		
Education		
Primary/Elementary School	10	45.4
Secondary School	6	27.3
Tertiary Institution	6	27.3
Years of service		
<1	2	9.1
1–5	10	45.4
6–10	6	27.3
11–15	2	9.1
>15	2	9.1
Have business license		
No	12	55.5
Yes	10	45.5
Service rendered		
Females	8	36.0
Males	7	32.0
Unisex	7	32.0
Number of worker(s) per shop		
1	21	95.5
2	1	4.5
Assistant(s) per shop		
None	20	91.0
1 person	1	4.5
2 people	1	4.5

For the beauty salon appearance, all salons impressively had proper lighting when measured with a lux meter, 21 (95.5 %) salons passed the heat stress evaluation, and 20 salons (91.0 %) passed the floor. 18 (81.8 %) salons passed the sound meter evaluation with a cut-off of 85 dBA; the other criteria for the hair salons visited 77.3 % had a proper indicating symbol of shop name and type of services rendered, but had no windows in good condition (72.7 %) due to damaged parts. Most passed the criteria for standard floors (90.9 %), walls (68.2 %), ceilings (68.2 %), and doors (90.9 %). For worker ergonomics and training, most did not report prolonged and continuous hand movements (81.8 %) or work characteristics (86.4 %) like standing, working, reaching, or raising for a prolonged period of time. However, only 18.2 % had valid worker training certificates regarding workplace sanitation, and 4.5 % had annual health check-ups and medical fitness certificates. When assessing the environmental conditions of the beauty salons, it was reported that the majority had improper ventilation channels (86.4 %) and there were no gender-segregated bathrooms (0 %). Most salons passed the standard for proper hazardous waste disposal (90.9 %) and available water supply (86.4 %). In assessing the accident prevention and safety conditions of the beauty salons, most met the inspecting standard needed for PPE usage (54.5 %), appropriate tool use and storage (90.9 %) and electric shock protection system (95.4 %). Also, only 4.5 % of the hair salons had worker uniforms and none had a first aid box. See Table 2.

**Table 2:** Assessing the beauty salon standards according to Department of Health guidelines.

Benchmark item	Description	Beauty salon assessment	
		Pass n (%)	Fail n (%)
General beauty salon appearance			
Proper indicating symbol	Presence of a clear indicating symbol installed for service clarity. This could be made of wood, glass, glazed tiles or any permanent object attached to the storefront for easy client recognition	17 (77.3)	5 (22.7)
Floor	Evenly levelled floor made of permanent, strong and smooth material that is easy to clean and/or non-slip with no cracks or potholes.	20 (90.9)	2 (9.1)
Wall	No wall stains or hanging items except hairstyle posters, type of services rendered or client-provided information, and must be placed appropriately to minimize damages or accidents	15 (68.2)	7 (31.8)
Ceiling	No stains or mouldy growth and made of permanent, strong and smooth material that is easy to clean. No hanging items except hairstyle posters, type of services rendered or client-provided information, and must be placed appropriately to minimize damages or accidents	15 (68.2)	7 (31.8)
Window	In good working condition with no damaged or detached parts. Dust-free, clean and assembled in its original appearance	6 (27.3)	16 (72.7)
Door	In good working condition with no damaged or detached parts. Not dirty, unsightly and assembled in its original appearance	20 (90.9)	2 (9.1)
Lighting	Proper light intensity no less than 200 lux measured at work level. No dimming, glare, flickering or reflection that could irritate the eye	22 (100)	0 (0)
Heat	Prolonged heat exposure from electrical appliances	21 (95.5)	1 (4.5)
Noise	Prolonged noise exposure from work tools	18 (81.8)	4 (18.2)

Worker ergonomics and training			
Prolonged hand movements	Prolonged and continuous use of tools for more than an hour	18 (81.8)	4 (18.2)
Standing, walking, reaching and raising	Prolonged and continuous work characteristics such as standing, walking, reaching, and raising the arms above shoulder level for more than an hour	19 (86.4)	3 (13.6)
Training certification	Valid worker training certification with regard to work facility sanitation	4 (18.2)	18 (81.8)
Annual health checks	Service providers have annual health checks with medical fitness certificates	1 (4.5)	21 (95.5)

Environmental condition of the beauty salons			
Ventilation	Presence of ventilation channels (such as windows, doors, air vents, etc.) that allow for airflow. In the case of a device for ventilation like an air conditioner or fan, it is visibly in good condition, dust-free, thoroughly and periodically cleaned and maintained	3 (13.6)	19 (86.4)
Proper hazardous waste disposal	Hazardous solid wastes such as sharps, spray cans, and needles are properly segregated from general solid wastes (like hair, plastic bags or papers) and discarded in non-pierce through durable, well labelled and sealed containers or waste bins made of hard plastics or metal	20 (90.9)	2 (9.1)
Water supply	Available clean and sufficient water supply for service use	19 (86.4)	3 (13.6)
Restrooms	Presence of clean and sanitary toilets that are clearly marked and in good condition. Gender-segregated restrooms are vividly marked	0 (0)	22 (100)

Accident prevention and safety conditions			
Personal protective clothing (PPE)	Use of clean and personal protective clothing by both beauticians and clients. PPE should be in good condition, regularly disinfected, and smell and stain-free	12 (54.5)	10 (45.5)
Worker uniforms	Service providers must wear a distinct set of uniforms that is white or lightly coloured. It should be in good condition, regularly disinfected, clean, well organized and properly stored	1 (4.5)	21 (95.5)
Tools use and storage	Single-use tools and sharps are not reused and properly discarded as hazardous wastes. Others are properly and regularly cleaned and disinfected with chemicals (like 70 % ethyl alcohol), autoclaved, after each customer usage and stored in an orderly appropriate storage place	20 (90.9)	2 (9.1)
Electric shock protection system	Electric circuit within the salon is grounded and has an automatic disconnector with a power cut-off	21 (95.5)	1 (4.5)
First aid box	Available first aid box containing essential first aid items such as bandages, cotton swabs, plaster and over-the-counter remedies	0 (0)	22 (100)

Results obtained during the COVID-19 pandemic for the required precautionary measures taken by hair salons to avert the spread of the infectious disease show that majority of the salons did not effectuate the guidelines put in place by the Department of Health while providing services to clients. Measures such as the use of a registration or attendance platform for contract tracing, allocating no more than 2hrs for client services, instructing client use of face masks or face shields, and thoroughly sterilizing plastic tools with alcohol or a sterilization cabinet was not performed at any of the beauty salons. Only 1 (4.5 %) salon had an available online payment platform to minimize contact between hairdressers and clients, so also the use of a digital thermometer for temperature checks of clients before entering the salon. A couple of salons (9.1 %) had clients booking service appointments to reduce congestion, conducted regular tool sterilizations and disinfection; and properly disposed of their well-segregated wastes daily. The practice of handwashing before and after every client service and use of gloves, face masks/shields by hairdressers was done by 3 (13.6 %) beauty salons. It was reported that 4 (18.2 %) hair salons had a clearly defined area to maintain some distance between clients, 5 (22.7 %) had available hand wash or sterilization for clients, 7 (31.8 %) had towels used once per client after which it is washed and disinfected and 9 (40.9 %) cleaned and disinfected furniture used by clients to prevent contamination. See Table 3.

**Table 3:** Preventive measures that should be observed by beauty salons during the COVID-19 epidemic according to Department of Health guidelines.

Benchmark item	Description	Assessment	
		Pass n (%)	Fail n (%)
Attendance list	A registration platform that allows for contact tracing by the government during the COVID-19 pandemic	0 (0)	22 (100)
Appointment booking	A platform for clients to book an appointment for services in advance to reduce congestion in the waiting area of the beauty salons	2 (9.1)	20 (90.9)
Online payment	To minimize contact between beauticians and clients, a secure online payment system should be available	1 (4.5)	21 (95.5)
Client time allocation	Not spending more than 2 h per client to minimize time spent between beauticians and their clients	0 (0)	22 (100)
Temperature check	Using a digital thermometer, clients' temperatures are checked at the entrance to ensure a temperature below 37.5 °C	1 (4.5)	21 (95.5)
Client handwashing or sanitisation	Available sink or handwash basin with soap in good condition and clean for clients. Also, alcohol-based hand gel	5 (22.7)	17 (77.3)
Handwashing before and after every client service by hairdressers	Before and after servicing each client, hairdressers are to wash their hands to prevent contamination and spread of the disease	3 (13.6)	19 (86.4)
Use of face masks/shields by clients	Instructing mandatory use of face mask or shield (except while working on a client's face) during visits by the clients	0 (0)	22 (100)
Use of gloves, face masks/shields by hairdressers	All service providers wear gloves, face masks or shields for the entire work duration and are changed regularly	3 (13.6)	19 (86.4)
Hairdresser COVID-19 checks every fortnightly	The service provider does regular COVID-19 checks to be medically fit and symptom-free	19 (86.4)	3 (13.6)
Designated hair service area	A clearly defined area for styling, washing or other activities with well-marked chairs to keep at least 1 m distance between clients	4 (18.2)	18 (81.8)
Furniture covering	Each chair used by the client is in good condition, cleaned and draped with a disposable covering (table paper) after every client's use	19 (86.4)	3 (13.6)
Furniture sanitisation	Hair wash basin and shampoo bed in good condition, cleaned and disinfected after every use to prevent contamination and spread of the disease	9 (40.9)	13 (59.1)
Clean towels	Towels should be white or light coloured, used once for only one client. After use, it is washed, disinfected and stored properly in a well-organized area	7 (31.8)	15 (68.2)
Regular sterilization of tools	Work tools such as clippers, scissors, and picks are thoroughly disinfected either by autoclaves, sterilization cabinets or chemically with 70 % ethyl alcohol after each client's use	2 (9.1)	20 (90.9)
Clean plastic tools	Plastic tools such as combs, brushes, hairpins, curling irons etc. are in good condition, not damaged and are thoroughly disinfected either by autoclaves, sterilization cabinets or chemically with 70 % ethyl alcohol after each client's use	0 (0)	22 (100)
Proper waste disposal	Garbage is disposed of daily. Hazardous wastes such as sharps, spray cans, and needles are properly segregated from general solid wastes (like hair, plastic bags or papers) and discarded in non-pierce through durable, well labelled and sealed containers or waste bins made of hard plastics or metal	2 (9.1)	20 (90.9)

The precautionary measures that were mostly practised by the hair salons (86.4 %) were regular COVID-19 checks for the hairdressers and the draping of furniture with disposable covering for every client.

## 4 Discussion

To ensure the successful control of the COVID-19 pandemic in response to the first wave in March 2020, the Thai government introduced universal control measures like curfews, wearing face masks, soap hand washing or disinfecting with alcohol, avoidance of outdoor gatherings, restricted interstate travelling, social distancing and ultimately lockdowns [21]. A few months after the first wave in a bid to return to a normal situation, restaurants, markets, supermarkets, hair salons and other small businesses were permitted to re-open but not without observing precautionary guidelines introduced by the Ministry of Public Health. The poor compliance generally observed in this study could be due to social and economic hardships suffered from the first wave of the pandemic. Although the northeastern region of Thailand is said to be the most vulnerable having the highest poverty rate of about 13 % [22], as of January 2021, the region had the minority of localized COVID-19 cases in the entire Kingdom [23]. Reports suggest that the stringent measures and policies enacted by the Thai government to check the spread of COVID-19 infection came with business disruptions, financial and mental stress (such as income loss and suicidal tendencies) as well as social health problems [14,22]. However, to facilitate public health services and improve the quality of life in Thailand, the government issued measures to speed up the disbursement of compensation payments to eligible people and affected groups and also plans to lower the cost of living [24].

Due to occupational hazards, hairdressers are exposed to several health risks and this study is one of the very few studies that highlights and assesses the community-based policies observed during the COVID-19 pandemic in a semi-urban district in the northeastern region of Thailand. The mean age of our respondents was 41.8 (±8.2) years and more than half are females perhaps because compared to males, females are more concerned about their physical appearance as some do believe that beauty is part of a healthy physical state. As per the standards of the beauty salons, most items on the assessment criteria were passed: 100 % lighting, 95.5 % heat, and 81.8 % noise of general salon appearance items, except that 0 % of accident prevention and safety, environmental, had a first aid box and restrooms (no gender-segregated bathrooms), respectively. Furthermore, about 4.5 % of the beauty salon workers had annual health check-ups and work uniforms, and 18.2 % had training certificates regarding workplace sanitation. Results observed regarding the implementation of the guidelines and policies instructed by the Thailand Department of Health suggest non-compliance by most beauty salons. Most benchmark items were failed by the hair salons except for regular COVID-19 checks for the hairdressers and cleaning and draped furniture with disposable coverings for every client, which had an 86.4 % compliance rate.

An important finding from our study reports that the majority of the hair salons were health-conscious enough to conduct regular COVID-19 checks and use disposable furniture drapings to prevent transmission. This behaviour could be promoted by the municipal office in the area to encourage prevention as an environmental health officer, whereas the annual health evaluation for hairdressers did not pass, perhaps due to the COVID-19 situation, which resulted in lower participation in health checks because of concerns about infection in the hospital. Meanwhile, Thailand has a strong healthcare system that has provided universal health coverage to all Thais since 2002 [25].

The safety of staff, clients, and the public is of crucial concern in every workplace as it contributes to the success of the enterprise. In the study area, most hairdressing salons tend to be small and independently owned outlets prompting them to be just as safe as the owner makes it. However, there is ample concern for sanitation and hygiene issues in the entire beauty industry regarding infectious disease transmission [26,27]. Observations from our study show the very low acquisition (by salons) of training certificates for workplace sanitation and hygiene. Salon services require a good amount of tools and equipment, so staff should be properly trained on the hygienic and maintenance aspects of their use. This calls for a strategy that allows regular inspections according to stipulated health and environmental health standards. The Thai Department of Health should establish inspectorate divisions at the sub-district level that regularly monitor and ensure.

Given the challenges faced by beauty salons in adhering to the COVID-19 prevention checklist, it is essential to recognize the potential impact of financial strain resulting from the economy's complete shutdown. This, in turn, has raised concerns about the survival of many businesses. To address these issues and support economic recovery, the federal government should consider implementing measures such as tax relief, business loans, subsidies, compensation, and other forms of financial assistance. Overall, it can be asserted that the government has effectively managed to control the transmission of COVID-19 through the various measures, guidelines, and policies it has implemented since the onset of the pandemic.

The study adds valuable information about the COVID-19 virus situation in sub-district-level hair salons. However, there are several limitations. Only 22 beauty salons in the sub-district were selected to participate in the study. To keep the survey simple and quick during the pandemic, the research team sought no information on the serial number and certificate of calibration. The nature of the survey tool may not be clear and concise. In our hope to return to a new normal by achieving a ‘zero COVID’ target, business enterprises such as beauty salons should implement and strictly adhere to guidelines according to Department of Health standards. To motivate hairdressers' compliance with health and safety standards, training or education sessions regarding the prevention of infectious disease transmission should be conducted. Further research should also be done on the behaviours associated with health risks in hair salons at several levels, a provincial, national, or border-nation level.

## Ethical approval

Ethical approval for the study protocol was obtained from the Ethics Committee of Kasetsart University with the approved number KUCSC-HE-62-015. All the information obtained was guaranteed anonymous and participants’ privacy and confidentiality during and following completion of the study were duly ensured.

## Author contributions (use CRediT terms)

Conceptualization: Chonyitree Sangwijit, Nitikorn Phoosuwan.
Data curation: Chonyitree Sangwijit, Nitikorn Phoosuwan.
Formal analysis: Chonyitree Sangwijit, Nitikorn Phoosuwan, Fatima Ibrahim Abdulsalam.
Investigation: Chonyitree Sangwijit, Nitikorn Phoosuwan, Fatima Ibrahim Abdulsalam.
Methodology: Chonyitree Sangwijit, Nitikorn Phoosuwan.
Project administration: Chonyitree Sangwijit, Nitikorn Phoosuwan.
Resources: Chonyitree Sangwijit, Nitikorn Phoosuwan, Fatima Ibrahim Abdulsalam.
Software: Nitikorn Phoosuwan.
Validation: Chonyitree Sangwijit, Nitikorn Phoosuwan, Fatima Ibrahim Abdulsalam.
Visualization: Nitikorn Phoosuwan, Fatima Ibrahim Abdulsalam.
Writing – original draft: Chonyitree Sangwijit, Nitikorn Phoosuwan.
Writing – review & editing: Nitikorn Phoosuwan, Fatima Ibrahim Abdulsalam.

## Funding

There is no funding reported in this study.

## Availability of data material

The datasets used in the current study are available from the corresponding author on reasonable request.

## Declaration of competing interest

The authors declare that they have no known competing financial interests or personal relationships that could have appeared to influence the work reported in this paper.
